# Early GCase activity is a predictor of long-term cognitive decline in Parkinson’s disease

**DOI:** 10.1186/s40035-023-00373-x

**Published:** 2023-08-28

**Authors:** Linn Oftedal, Johannes Lange, Kenn Freddy Pedersen, Aleksander Hagen Erga, Ingvild Dalen, Ole-Bjørn Tysnes, Guido Alves, Jodi Maple-Grødem

**Affiliations:** 1 Centre for Movement Disorders, Centre for Brain Health, Stavanger University Hospital, 4011 Stavanger, Norway; 2 Department of Chemistry, Bioscience and Environmental Engineering, University of Stavanger, 4021 Stavanger, Norway; 3 Department of Neurology, Stavanger University Hospital, 4011 Stavanger, Norway; 4 Department of Social Studies, University of Stavanger, 4021 Stavanger, Norway; 5 Section of Biostatistics, Department of Research, Stavanger University Hospital, 4011 Stavanger, Norway; 6 Department of Clinical Medicine, University of Bergen, 5021 Bergen, Norway; 7 Department of Neurology, Haukeland University Hospital, 5053 Bergen, Norway

Dementia is a serious complication for many patients with advanced Parkinson’s disease (PD) and its incidence is increased in PD patients harbouring a mutation in *GBA1* [1]. However, cognitive impairment is not limited to late disease stages and can also manifest in patients with early untreated disease, leading to diminished social function and increased disability and caregiver burden. We have previously shown that reduced activity of the *GBA1* protein product, the lysosomal enzyme glucocerebrosidase (GCase), is linked with long-term progression to PD dementia [2]. In this study, we extend this by determining the association of cerebrospinal fluid (CSF) GCase activity with the development of cognitive impairment through the analysis of neuropsychological test data collected over 10 years of prospective follow-up of patients with incident PD.

A total of 117 patients from the Norwegian ParkWest study [3] with GCase activity measured in CSF samples taken at the time of PD diagnosis (median delay, 38 days) [4] were included in the study (Additional file 1: Table S1). Neuropsychological testing assessed functions in four cognitive domains: attention, executive function, verbal learning and memory, and visuospatial skills. A score for each domain was calculated by taking the average of the test scores after conversion into the Percent of Maximum Possible scores as described in Additional file 2: Methods.

To assess the association of reduced baseline GCase activity with the change in cognitive function over the first 10 years of PD (median follow-up, 9.0 years [interquartile range, 1.0]), we applied linear mixed-effects models. The patients were stratified into tertiles based on the level of CSF GCase activity and those with the highest activity (> 1.12 mU/mg) were compared to the group with medium (0.80–1.12 mU/mg) or low GCase activity (< 0.80 mU/mg). At the time of PD diagnosis, there was no significant difference in the MMSE scores across the three GCase activity groups (Fig. 1a; Additional file 1: Table S2). However, both the medium (β =  − 1.58 transformed points; 95% confidence interval [CI] − 2.91 to − 0.25, *P* = 0.022) and the low GCase activity groups (β =  − 2.26 transformed points; 95%CI − 3.59 to − 0.94, *P* = 0.001) were predicted to experience a faster annual decline in MMSE score, compared to the high activity group. Over the 10 years of the study, patients in the high GCase activity group were not predicted to experience a significant decline in MMSE score (*P* = 0.159), whereas those in the medium- or low-activity group were estimated to decline from about 29 to 26 or to 24 MMSE points, respectively.

**Fig. 1:**
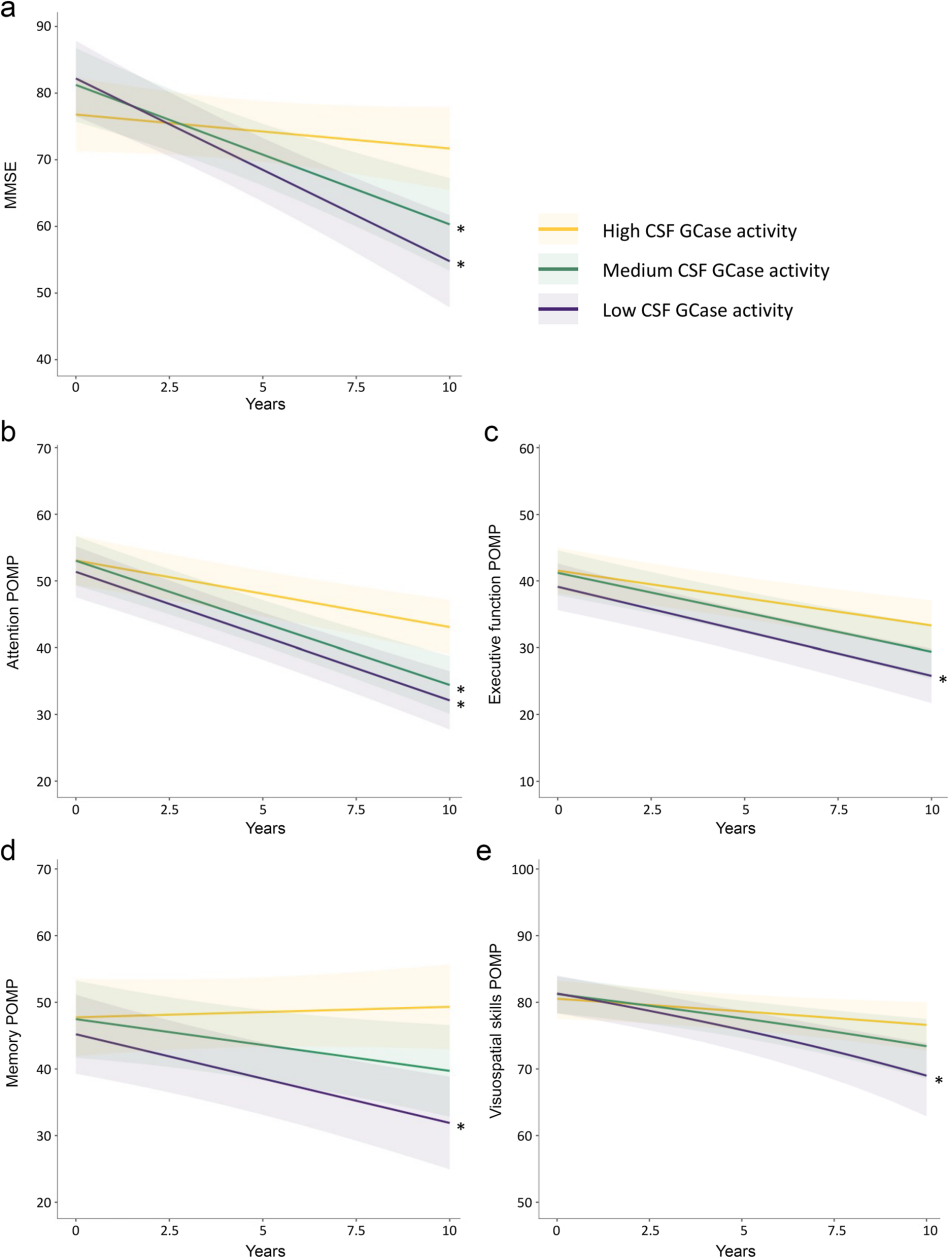
Prediction of changes of scores measuring cognitive impairment over time. Patients (*n* = 117) were grouped by GCase activity level (high, medium, and low). **a** MMSE scores, and POMP scores for **b** attention, **c** executive function, **d** memory, and **e** visuospatial skills. MMSE scores were transformed before plotting as described in the Methods. *Significant difference from the high GCase activity group (*P* < 0.05). *POMP* percent of maximum possible

At the baseline visit, there was no significant difference in the performance in tests assessing attention, executive function, verbal learning and memory, or visuospatial skills between the GCase activity groups (Additional file 1: Table S2). Assessment of the annual change in performance in individual cognitive domains showed that the low activity group was predicted to decline faster in scores of attention (β =  − 0.92; 95%CI − 1.58 to − 0.28, *P* = 0.006), executive function (β =  − 0.61; 95%CI − 1.20 to − 0.03, *P* = 0.042), memory (β =  − 1.60, 95%CI − 2.63 to − 0.58, *P* = 0.003), and visuospatial skills (β = − 0.84 transformed points, 95%CI − 1.63 to − 0.06, *P* = 0.038) (Fig. 1b–e; Additional file 1: Table S2). However, the group with the medium level of GCase activity only declined significantly faster than the high activity group in measures of attention (β =  − 0.95; 95%CI − 1.60 to − 0.30; *P* = 0.005), and for the other three cognitive domains studied the difference in the annual rate of change was not significant (all *P* > 0.05). The results were largely unchanged when the analysis was repeated including only the 105 PD patients without *GBA1* mutations (Additional file 2: Methods and Additional file 1: Fig. S1 and Table S2). Specifically, we found that the size of the effect and significance of the association of GCase activity status with the decline in cognitive function remained comparable apart from the scores for executive function, for which there was no significant difference in the annual decline between the high and the low activity groups (*P* = 0.086).

In the brains of patients with PD, GCase dysfunction is paralleled by α-synuclein accumulation [5, 6]. The mild negative correlation between α-synuclein pathology and GCase activity may be involved in the increased cognitive decline observed in the patients with the lowest GCase activity in this study. However, how GCase dysfunction influences PD pathology (and vice versa) is unclear. Most *GBA1* mutation carriers do not develop PD [7, 8], suggesting that the relationship is not causative. Recent findings from animal and cell models support that lower GCase activity levels do not initiate α-synuclein aggregation, but promote α-synuclein aggregation depending on the physiological state of the extant α-synuclein [9, 10], and factors beyond GCase depletion play a role in influencing the incidence of the disease.

GCase is a promising target for the treatment of neurodegenerative disorders caused by the progressive aggregation of α-synuclein. A key challenge in developing these therapies is identifying a defined testing window where such interventions would have a maximum impact. At the time of PD diagnosis, the patients in the three GCase activity groups had comparable performance and those patients in the low activity group who were cognitively intact could represent a high-risk population to test GCase-targeting and neuroprotective therapies. Crucially, this could provide a means by which to recruit patients from the idiopathic PD population (i.e., those without a *GBA1* mutation) to pivotal clinical trials that might otherwise prioritise *GBA1*-PD patients.

We have recently shown that the GCase dysfunction at the time of PD diagnosis is linked to an increased risk of later development of dementia [2]. Although dementia is an important milestone in a clinical setting, it is less suitable as an outcome in clinical trials for modification of cognitive decline as dementia often requires many years of follow-up and/or very large cohorts to capture a meaningful number of events. For trial design, this is compounded as dementia is commonly a late-stage event, and potentially disease-modifying drugs are thought to be most effective when initiated early in the disease course. By contrast, cognitive decline is a gradual process that starts early in a substantial subset of patients and is frequently included as an outcome in PD clinical trials. Therefore, we performed a power calculation to estimate the predicted benefit (concerning trial size) of limiting enrolment to a clinical trial to patients in the lowest tertile of GCase activity, compared to a design in which all newly diagnosed patients are eligible for trial inclusion (an “all-comer” design) (Additional file 2: Methods). The trial was for a hypothetical therapeutic agent that stopped the decline of total MMSE score. We found that enriching a three-year trial for patients with low GCase activity reduced the required sample size for the trial by 2.5-fold compared with an equally powered trial without. In one example, a trial designed to have 80% power to observe the primary outcome would necessitate the recruitment of 658 “all-comers” with early PD, whereas only 266 low-GCase-activity participants would be needed for the same power (Additional file 1: Fig. S2).

Our study had some limitations. We did not account for anxiety or depression, which can affect performance in cognitive test batteries and have been linked to *GBA1* in other populations. Further, we did not account for other pathological changes such as co-morbid Alzheimer’s disease and neuroinflammation, which are reported risk factors for cognitive impairment in PD [11]. Our study had also several strengths, including the recruitment of incident, unselected patients that are representative of the PD population of the region, the analysis of CSF samples collected at the time of initial clinical diagnosis, the long follow-up time with repeated batteries of the same neuropsychological tests, and low attrition rates.

In conclusion, we found that low CSF GCase activity at the time of initial diagnosis is linked to a faster annual decline in clinical scales measuring global cognition and specific cognitive domains over the first 10 years of PD. The link between GCase dysfunction and disease progression provides insight into the pathogenesis of the disease and novel perspectives for GCase-targeted therapies to prevent neurodegeneration, and could provide a valuable biomarker to identify patients at risk of more severe disease.

### Supplementary Information

**Additional file 1: Table S1.** Cohort overview and GCase activity at baseline. **Table S2.** Relationship between GCase activity status and predicted annual change in scores in tests measuring cognitive function estimated using linear mixed models. **Fig. S1.** Prediction of scores measuring cognitive impairment over time. **Fig. S2.** Reduced trial size in GCase-targeted clinical trials compared to a traditional “all-comer” design.

**Additional file 2: Methods.**

## Data Availability

The datasets used during the current study are not publicly available due to the condition of the study’s ethical approvals but are available from the corresponding author on reasonable request.

## Abbreviations

CI: Confidence interval
GCase: Glucocerebrosidase
MMSE: Minimal-Mental State Examinations
PD: Parkinson’s disease
