# Supplementary material for: Early GCase activity is a predictor of long-term cognitive decline in Parkinson’s disease
Source: Transl Neurodegener. 2023 Aug 28;12:41. doi: 10.1186/s40035-023-00373-x (PMC10463992; doi:10.1186/s40035-023-00373-x)
Supplement: Supplementary file 1 — Additional file 1: Table S1. Cohort overview and GCase activity at baseline. Table S2. Relationship between GCase activity status and predicted annual change in scores in tests measuring cognitive function estimated using linear mixed models. Fig. S1. Prediction of scores measuring cognitive impairment over time. Fig. S2. Reduced trial size in GCase-targeted clinical trials compared to a traditional “all-comer” design. [file 40035_2023_373_MOESM1_ESM.docx]

**Additional file 1**

**Supplementary Table S1. Cohort overview and GCase activity at baseline**

|  | All PD |  | **High GCase activity group** | **Medium GCase activity group** | **Low GCase activity group** |
| --- | --- | --- | --- | --- | --- |
| *n* | 117 |  | 39 | 39 | 39 |
| Age, years | 67.2 ± 9.5 |  | 63.7 ± 11.2 | 68.5 ± 8.2 | 69.4 ± 8.0 |
| Time from first symptoms, years | 2.2 ± 1.7 |  | 2.0 ± 1.6 | 2.2 ± 1.7 | 2.4 ± 1.9 |
| Sex M, *n* (%) | 76 (65%) |  | 20 (51.3) | 24 (61.5) | 32 (82.1) |
| Education, years | 11.2 ± 3.1 |  | 11.1 ± 3.1 | 11.0 ± 3.4 | 11.5 ± 2.9 |
| MMSE, median IQR | 29.0 ± 2.0 |  | 29.0 ± 2.0 | 28.0 ± 4.0 | 28.0 ± 2.0 |
| UPDRS III | 22.4 ± 10.6 |  | 18.6 ± 8.6 | 22.1 ± 11.1 | 26.3 ± 10.7 |
| H&Y, median IQR | 2.0 (0.8) |  | 1.5 ± 1.0 | 2.0 ± 1.0 | 2.0 ± 1.0 |
| GCase, mU/mg | 0.99 ± 0.37 |  | 1.41 ± 0.29 | 0.93 ± 0.09 | 0.63 ± 0.13 |

Values are presented as mean ± SD, unless stated.

Abbreviations: UPDRS III, Unified Parkinson’s Disease Rating Scale part III; MMSE, Minimal-Mental State Examinations; H&Y, Hoehn and Yahr.

**Supplementary Table S2. Relationship between GCase activity status and predicted annual change in scores in tests measuring cognitive function estimated using linear mixed models.**

|  | ***All PD, n = 117*** | | | |  | ***Idiopathic PD, n = 105*** | | | |
| --- | --- | --- | --- | --- | --- | --- | --- | --- | --- |
| **Scores** | **Main effect^a^ β (95% CI)** | ***P*** | **Interaction with time^a^ β (95% CI)** | ***P*** |  | **Main effect^a^ β (95% CI)** | ***P*** | **Interaction with time^a^ β (95% CI)** | ***P*** |
| MMSE ^b^ |  |  |  |  |  |  |  |  |  |
| Age at baseline, years | -0.65 (-1.54 to -0.24) | <0.001 |  |  |  | -0.63 (-0.91 to -0.35) | <0.001 |  |  |
| Sex | 4.59 (-0.28 to 9.46) | 0.067 |  |  |  | 6.42 (1.20 to 11.64) | 0.018 |  |  |
| Education, years | 1.04 (0.28 to 1.81) | 0.009 |  |  |  | 1.13 (0.30 to 1.97) | 0.009 |  |  |
| Medium GCase activity ^c^ | 3.24 (-2.90 to 9.13) | 0.282 | -1.58 (-2.91 to -0.25) | 0.022 |  | 3.63 (-2.67 to 9.92) | 0.262 | -1.76 (-3.13 to -0.40) | 0.013 |
| Low GCase activity ^c^ | 3.34 (-2.80 to 9.48) | 0.289 | -2.26 (-3.59 to -0.94) | 0.001 |  | 4.29 (-2.35 to 10.92) | 0.209 | -2.36 (-3.75 to -0.96) | 0.001 |
| Executive function |  |  |  |  |  |  |  |  |  |
| Age at baseline, years | -0.68 (-0.87 to -0.49) | <0.001 |  |  |  | -0.65 (-0.85 to -0.45) | <0.001 |  |  |
| Sex | 3.99 (-0.87 to 7.49) | 0.067 |  |  |  | 3.58 (-0.12 to 7.28) | 0.061 |  |  |
| Education, years | 0.82 (0.27 to 1.37) | 0.004 |  |  |  | 0.73 (0.14 to 1.33) | 0.017 |  |  |
| Medium GCase activity ^c^ | -0.49 (-4.50 to 3.52) | 0.812 | -0.49 (-1.08 to 0.09) | 0.103 |  | -0.89 (-5.12 to 3.35) | 0.683 | -0.51 (-1.14 to 0.11) | 0.111 |
| Low GCase activity ^c^ | -3.2 (-7.45 to 0.97) | 0.135 | -0.61 (-1.20 to -0.03) | 0.042 |  | -3.52 (-8.01 to 0.97) | 0.128 | -0.57 (-1.21 to 0.07) | 0.086 |
| Attention |  |  |  |  |  |  |  |  |  |
| Age at baseline, years | -0.64 (-0.86 to -0.43) | <0.001 |  |  |  | -0.61 (-0.84 to -0.38) | <0.001 |  |  |
| Sex | 8.36 (4.43 to 12.30) | <0.001 |  |  |  | 8.14 (3.85 to 12.44) | <0.001 |  |  |
| Education, years | 0.60 (-0.02 to 1.22) | 0.061 |  |  |  | 0.55 (-0.14 to 1.24) | 0.120 |  |  |
| Medium GCase activity ^c^ | -0.42 (-4.99 to 4.14) | 0.856 | -0.95 (-1.60 to -0.30) | 0.005 |  | -0.64 (-5.60 to 4.32) | 0.801 | -1.03 (-1.70 to -0.36) | 0.004 |
| Low GCase activity ^c^ | -2.74 (-7.54 to 2.06) | 0.265 | -0.92 (-1.58 to -0.28) | 0.006 |  | -2.05 (-7.31 to 3.21) | 0.446 | -1.05 (-1.75 to -0.36) | 0.004 |
| Visuospatial ^b^ |  |  |  |  |  |  |  |  |  |
| Age at baseline, years | -0.69 (-0.92 to -0.47) | <0.001 |  |  |  | -0.70 (-0.94 to -0.50) | <0.001 |  |  |
| Sex | -0.91 (-5.02 to 3.20) | 0.666 |  |  |  | -0.77 (-5.22 to 3.69) | 0.734 |  |  |
| Education, years | -0.03 (-0.67 to 0.62) | 0.936 |  |  |  | -0.01 (-0.72 to 0.71) | 0.985 |  |  |
| Medium GCase activity ^c^ | 1.08 (-3.98 to 6.14) | 0.678 | -0.53 (-1.30 to 0.25) | 0.185 |  | 1.33 (-4.15 to 6.80) | 0.636 | -0.57 (-1.38 to 0.23) | 0.167 |
| Low GCase activity ^c^ | 0.92 (-4.36 to 6.21) | 0.733 | -0.84 (-1.63 to -0.06) | 0.038 |  | 1.15 (-4.62 to 6.93) | 0.695 | -0.97 (-1.80 to -0.14) | 0.024 |
| Memory |  |  |  |  |  |  |  |  |  |
| Age at baseline, years | -0.94 (-1.29 to -0.60) | <0.001 |  |  |  | -0.87 (-1.22 to -0.51) | <0.001 |  |  |
| Sex | 8.84 (2.48 to 15.20) | 0.007 |  |  |  | 9.59 (2.92 to 16.25) | 0.006 |  |  |
| Education, years | 1.00 (0.00 to 2.01) | 0.052 |  |  |  | 1.31 (0.23 to 2.38) | 0.019 |  |  |
| Medium GCase activity ^c^ | -0.11 (-7.69 to 7.47) | 0.978 | -0.95 (-1.98 to 0.074) | 0.072 |  | -0.06 (-7.90 to 7.78) | 0.989 | -1.18 (-2.23 to -0.13) | 0.030 |
| Low GCase activity ^c^ | -2.91 (-10.82 to 5.01) | 0.473 | -1.60 (-2.63 to -0.58) | 0.003 |  | -1.38 (-9.64 to 6.88) | 0.744 | -1.98 (-3.05 to -0.91) | <0.001 |

^a^ Models adjusted for sex, age, and years of education at baseline. The main effect indicates the effect of carrier status on the intercept and the interaction with time indicates the effect of carrier status on the slope (change in value per year) of the model.

^b^ The MMSE and visuospatial skills scores were transformed before analysis as described in Additional file 2: Methods

^c^ ref is CSF GCase high activity group (>1.12 mU/mg) compared to medium GCase activity (0.80 to 1.12 mU/mg) and low GCase activity (<0.80 mU/mg)

Abbreviation: CI, confidence interval; MMSE, Mini-mental state examination.


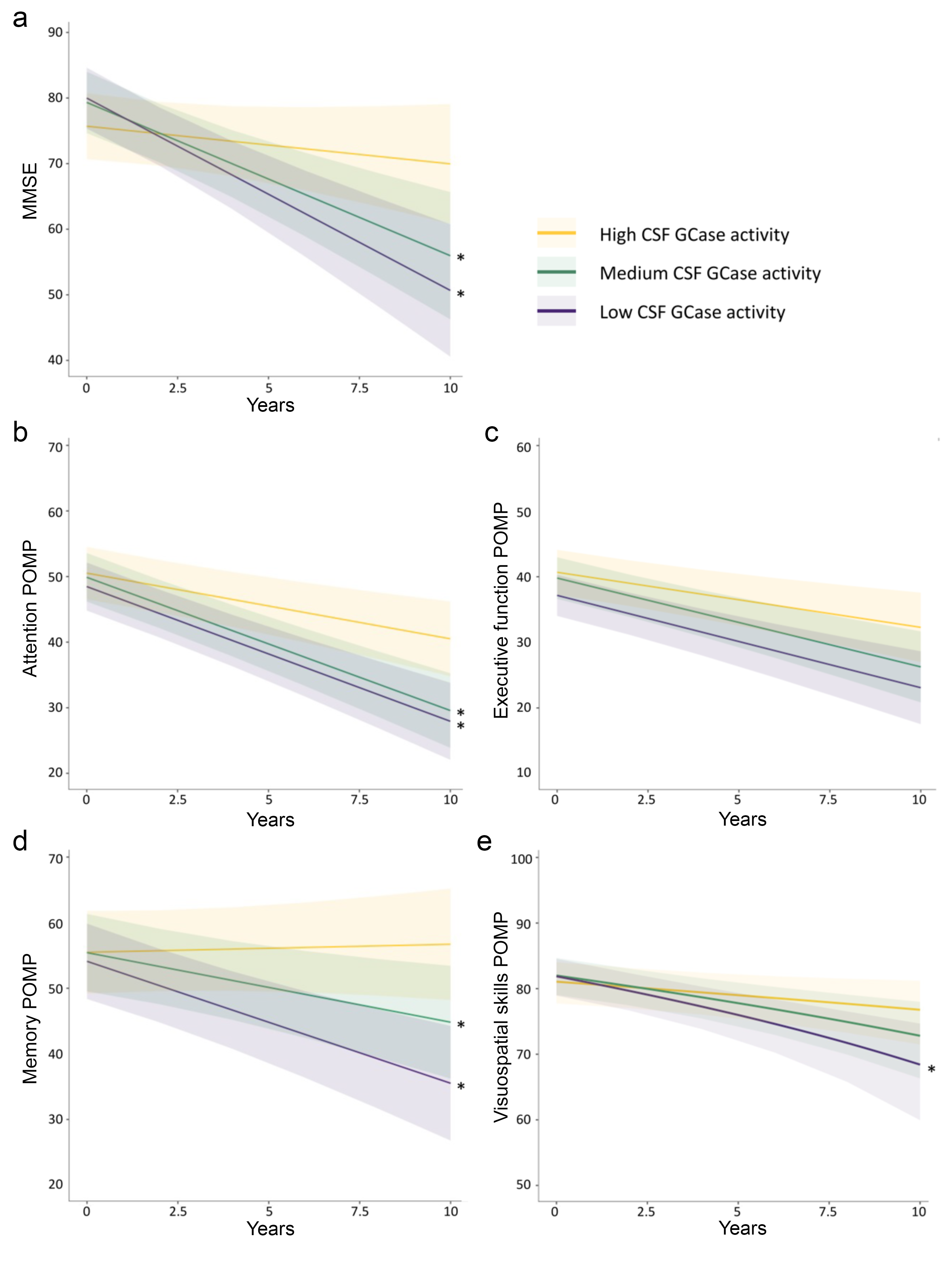


**Fig. S1.** Prediction of scores measuring cognitive impairment over time. Idiopathic patients (*n* = 105) grouped by GCase activity level (high, medium, low as outlined in the figure key). (**a**) MMSE scores, and POMP scores for (**b**) attention, (**c**) Executive function, (**d**) memory, and (**e**) visuospatial skills. MMSE scores were transformed before plotting as described in the Methods.

* Significant difference from the reference group (high GCase activity) (*P* < 0.05).

**
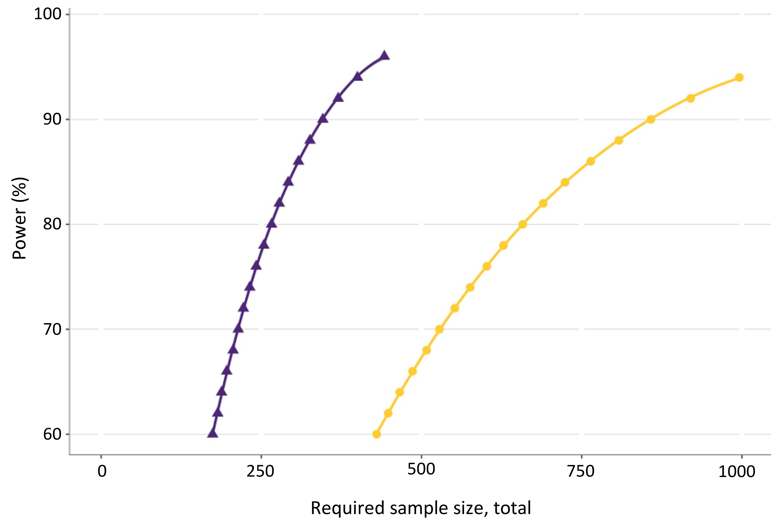
**

**Fig. S2. Reduced trial size in GCase-targeted clinical trials compared to a traditional “all-comer” design.** The required sample size for clinical trials enrolling only those patients within the lowest tertile of GCase activity (purple triangles) or an “all-comer” design with nonselected patients with PD (yellow circles) across varying levels of power to detect a between–within subjects interaction effect.
