# Supplementary material for: Early GCase activity is a predictor of long-term cognitive decline in Parkinson’s disease
Source: Transl Neurodegener. 2023 Aug 28;12:41. doi: 10.1186/s40035-023-00373-x (PMC10463992; doi:10.1186/s40035-023-00373-x)
Supplement: Supplementary file 2 — Additional file 2: Methods. [file 40035_2023_373_MOESM2_ESM.docx]

**Additional File 2**

**METHODS**

**Design and setting**

Patients were recruited from the Norwegian ParkWest study of incident PD in Southwestern Norway [1]. Only those with a confirmed clinical diagnosis of PD according to the UK brain bank criteria [2] at their latest or final clinical visit or pathological confirmation (if post-mortem examination was performed) were included. Of 190 participants, cerebrospinal fluid (CSF) was available from 120 participants consenting to lumbar puncture (LP) at study entry. *GBA* mutation carrier status was available for all and has been summarised by Lunde et al [3]. Five nonsynonymous variants were detected by whole exome sequencing (rs76763715/N370S, n = 1; rs75548401/T369M, n = 5; rs2230288/E326K, n = 14; rs369068553/V460L, n = 1; and rs781152868/Y135C, n = 1) [4] and rs421016/L444P (n = 1) was obtained by restriction fragment length polymorphism assays [3].

**Clinical assessment**

A standardized examination program was administered at study entry, including Unified Parkinson’s Disease Rating Scale (UPDRS) part III) [5] and Hoehn and Yahr scale [6]. Further, a program of cognitive tests was administered by trained study nurses at baseline, year one, and every two years thereafter. Global cognition was assessed with the Mini-Mental State Examination (MMSE) [7]. Neuropsychological testing assessed functions in four cognitive domains: (1) attention (Stroop word reading and colour naming test) [8]; (2) executive function (Semantic Verbal Fluency Test [9] and Stroop interference condition test); (3) verbal learning and memory (California Verbal Learning Test II [10], from which total words immediate recall, short-delay, and long-delay free recall scores were included); and (4) visuospatial skills (Visual Object and Space Perception Battery Silhouettes and Cube subtests) [11]. A score for each of the four domains was calculated by taking the average of the test scores after conversion into Percent of Maximum Possible (POMP) scores [12].

**GCase activity measurements in CSF**

The level of GCase activity was available for 117 participants (3 of 120 were excluded for technical reasons) [13]. Briefly, LP and sample treatment was conducted according to standardized procedures [14]. CSF samples (diluted 1:2) were added to the substrate 4-methylumbelliferyl β-D-glucopyranoside and incubated for three hours incubation at 37°C. After stopping the reaction by adding 0.2 M glycine pH 10.2, the concentration of the fluorescent cleavage product, 4-methylumbelliferyl, was measured (Excitation: 360 nm/Emission: 446 nm). One unit (U) of GCase activity was defined as the amount of enzyme that hydrolyses 1 nmol of substrate/min at 37°C and expressed as mU/mg of total protein concentration [15].

**Statistical analysis**

Between-group differences in baseline demographics and clinical variables were assessed using independent samples t-tests, Mann-Whitney U tests, or χ^2^-tests as appropriate. Adjusted analyses were conducted using multiple linear regression with age and sex as covariates.

Linear mixed effects analyses performed in R and *lme4* [16] were utilized to determine the relationship between GCase activity and the progression of cognitive impairment. The MMSE and visuospatial skills domain scores were transformed before modelling: MMSE scores were transformed as described by Philipps et al. [17] to minimize bias due to the ceiling/floor effect and curvilinearity of the raw MMSE score. Visuospatial skills domain scores were transformed by power transformation with parameter λ=3.8 [12]. The time of the repeated measures was computed in years from the baseline date and included as a fixed effect. The interaction between time (years) and GCase activity tertile was included as a fixed effect. All models had patients’ IDs as random intercepts and random slope of time. Analyses were performed unadjusted and adjusted for sex, age and education (in years) at baseline. The size of the effect of GCase activity status or the level of significance on the outcome did not substantially differ between the unadjusted and adjusted models (data not shown). Normality of residuals was verified by Q-Q plots and homoscedasticity and linearity visually assessed. For visualisation of the results, predictions were generated for a male of average age and education and the visuospatial skills scores were back transformed before plotting. Primary analyses were corrected for multiple comparisons using the Benjamini–Hochberg false discovery rate (FDR) method at FDR < 0.05.

**Power calculations for the clinical trial**

Power calculations were performed to compare two scenarios, modelling hypothetical clinical trials that either considered all newly diagnosed patients with PD eligible (“all-comers”) for enrolment or that limited eligibility to those in the lowest tertile of GCase activity. For the “placebo group” the expected trajectories were extracted from population averaged models adjusted for age, sex, and education (for all patients (-1.74) or only the “low GCase activity group” (-2.83)). The covariance matrices were based on separate estimates of both variance and correlation (respectively, 362 and 0.60 and 367 and 0.57). The hypothetical trial was designed to last 3 years and the intervention was presumed to stop progression measured using MMSE. The required sample sizes to obtain a power of between 60 to 96% to detect such differences in slopes (i.e., the between-within subjects’ interaction effect) at a 5% significance level were estimated using Stata function power repeated, which incorporates F tests with Greenhouse-Geisser correction for lack of sphericity.

**Additional File 2 References**

1. Alves G, Muller B, Herlofson K, HogenEsch I, Telstad W, Aarsland D, Tysnes OB, Larsen JP, Norwegian ParkWest study g: **Incidence of Parkinson's disease in Norway: the Norwegian ParkWest study**. *J Neurol Neurosurg Psychiatry* 2009, **80**(8):851-857.

2. Daniel SE, Lees AJ: **Parkinson's Disease Society Brain Bank, London: overview and research**. *J Neural Transm Suppl* 1993, **39**:165-172.

3. Lunde KA, Chung J, Dalen I, Pedersen KF, Linder J, Domellof ME, Elgh E, Macleod AD, Tzoulis C, Larsen JP *et al*: **Association of glucocerebrosidase polymorphisms and mutations with dementia in incident Parkinson's disease**. *Alzheimers Dement* 2018, **14**(10):1293-1301.

4. Gaare JJ, Nido GS, Sztromwasser P, Knappskog PM, Dahl O, Lund-Johansen M, Maple-Grodem J, Alves G, Tysnes OB, Johansson S *et al*: **Rare genetic variation in mitochondrial pathways influences the risk for Parkinson's disease**. *Mov Disord* 2018, **33**(10):1591-1600.

5. Fahn S: **Unified Parkinson's Disease Rating Scale**. *Recent Development in Parkinson's Disease* 1987.

6. Hoehn MM, Yahr MD: **Parkinsonism: onset, progression and mortality**. *Neurology* 1967, **17**(5):427-442.

7. Folstein MF, Folstein SE, McHugh PR: **"Mini-mental state". A practical method for grading the cognitive state of patients for the clinician**. *J Psychiatr Res* 1975, **12**(3):189-198.

8. Golden C.J. FSM: **The Stroop Color and Word Test. Wood Dale**. IL: Stoelting Co; 1998.

9. Benton AL, Hamsher, K.: **Multilingual Aphasia Examination. Manual of Intructions**, 2nd edn. Iowa City: AJA associates; 1989.

10. Delis D.C. KJH, Kaplan E., Ober B.A., : **California Verbal Learning**, 2nd edn. San Antonio, TX: Psychological Corp/Harcourt Assessment Inc; 2000.

11. Warrington EK, James M.: **The visual Object and Space Perception Battery**. England: Thames Valley Test Co; 1991.

12. Chung J, Ushakova A, Doitsidou M, Tzoulis C, Tysnes OB, Dalen I, Pedersen KF, Alves G, Maple-Grodem J: **The impact of common genetic variants in cognitive decline in the first seven years of Parkinson's disease: A longitudinal observational study**. *Neurosci Lett* 2021, **764**:136243.

13. Oftedal L, Maple-Grodem J, Dalen I, Tysnes OB, Pedersen KF, Alves G, Lange J: **Association of CSF Glucocerebrosidase Activity With the Risk of Incident Dementia in Patients With Parkinson Disease**. *Neurology* 2022.

14. Alves G, Lange J, Blennow K, Zetterberg H, Andreasson U, Forland MG, Tysnes OB, Larsen JP, Pedersen KF: **CSF Abeta42 predicts early-onset dementia in Parkinson disease**. *Neurology* 2014, **82**(20):1784-1790.

15. Oftedal L, Maple-Grodem J, Forland MGG, Alves G, Lange J: **Validation and assessment of preanalytical factors of a fluorometric in vitro assay for glucocerebrosidase activity in human cerebrospinal fluid**. *Sci Rep* 2020, **10**(1):22098.

16. Bates D, Mächler M, Bolker B, Walker S: **Fitting Linear Mixed-Effects Models Using lme4**. *Journal of Statistical Software* 2015, **67**:1–48.

17. Philipps V, Amieva H, Andrieu S, Dufouil C, Berr C, Dartigues JF, Jacqmin-Gadda H, Proust-Lima C: **Normalized Mini-Mental State Examination for assessing cognitive change in population-based brain aging studies**. *Neuroepidemiology* 2014, **43**(1):15-25.
